# Evaluation of *Trichoderma* spp. Isolates in Cocoa Seed Treatment and Seedling Production

**DOI:** 10.3390/plants10091964

**Published:** 2021-09-20

**Authors:** Willian Nogueira de Sousa, Nayane Fonseca Brito, Cristina Aledi Felsemburgh, Thiago Almeida Vieira, Denise Castro Lustosa

**Affiliations:** 1 Altamira City Hall, Altamira 68371-456, Brazil; 2 Institute of Biodiversity and Forests, Federal University of Western Pará, Santarém 68040-255, Brazil; nayanebrito4@gmail.com (N.F.B.); crisalefel@gmail.com (C.A.F.); thiago.vieira@ufopa.edu.br (T.A.V.)

**Keywords:** biocontrol, *Theobroma cacao*, biostimulant, germination, growth promotion

## Abstract

Isolates of *Trichoderma* spp., a soil fungus, has been used to control diseases and promote plant growth, reducing the use of chemicals in the production of seedlings of different plant species. We evaluated the effect of some *Trichoderma* spp. isolates on seed treatment and seedling production of *Theobroma* *cacao*. Five isolates from the Amazon region were tested. In laboratory, the following variables were evaluated for seed treatments: germination, germination speed index, radicle and hypocotyl lengths, and fungi incidence. In nursery, the following forms of application were tested: via seeds; in the substrate at pre-planting; monthly in post-planting substrate, and also their combination. The following was evaluated: height, diameter, number of leaves, root length, leaf area, and shoot dry mass and root system. Inoculation with *Trichoderma* increased the length of the radicle and hypocotyl and showed no fungi in the seeds. In seedlings, some treatments increased height and plant root dry mass. The use of *Trichoderma* was beneficial for seeds and appeared favorable for *T. cacao* production.

## 1. Introduction

Recently, an increase in the use of biological agents in agriculture has been observed [1,2], which consists of the application of microorganisms or their metabolites to protect seeds and promote germination and plant growth and management of different pathogens and pests [3,4]. Among the microorganisms that can be used, several isolates of *Trichoderma* spp. showed different mechanisms of action, such as antibiosis, mycoparasitism, competition, resistance induction, and growth promotion, in addition to solubilization of nutrients [5,6,7,8]. These properties provide productive and economic gains in the process of growing important crops.

Cocoa (*Theobroma cacao* L.) is a species of great economic and social importance globally. Worldwide, cocoa-producing areas occupy approximately 6.7 million hectares, with an estimated production of 4.7 million tons, generating a revenue of approximately 8.6 billion dollars per year [9]. In Brazil, this crop gained prominence with the expansion of chocolate consumption. Its cultivation represents a good example for composing agroforestry systems, as it can be cultivated under the shade of the thinned native forest (“cabruca” system) in association with other species, without the need of deforestation [10].

Pests and diseases affect cocoa crops strongly, constituting a major problem for producers [11]. The use of agrochemicals, applied from seed treatment and seedling preparation through cultivation to post harvest, has caused serious problems of environmental contamination, and therefore, products in favor of sustainable agriculture have been studied and developed.

The use of *Trichoderma* spp. on *T. cacao* has mainly been studied for disease control [12]. However, this fungus can also promote the development of seedlings and root systems in addition to delaying drought responses when plants are subjected to water stress [13]. Thus, research is important to identify *Trichoderma* isolates that are efficient and adapted to the environmental conditions of the producing region, which can be used for treating seeds and seedlings and in the development of cultivated plants.

The sanitary quality of seeds is essential for the production of healthy and vigorous seedlings [14] and for the production of quality, vigorous, and pest and disease-free seedlings with good adaptation and field growth [15]. Thus, the treatment of seeds and seedlings with *Trichoderma* spp. is an alternative that can generate good results in different plant species, as it is considered sustainable.

Our hypothesis was that the application of *Trichoderma* stimulates the germination of cacao seeds and the initial growth of seedlings, reducing the time the plants remain in the nursery, ensuring quality seedlings. In this article, we evaluate the effect of five *Trichoderma* isolates in different application modes on seed treatment and development of *T. cacao* seedlings.

## 2. Results

### 2.1. Trichoderma Effect on T. cacao Seed Germination

There was a significant difference for the radicle and hypocotyl length and the incidence of fungi. In all treatments, germination was greater than 95% (Table 1). *Trichoderma* did not influence germination speed compared to control.

**Figure 1:**
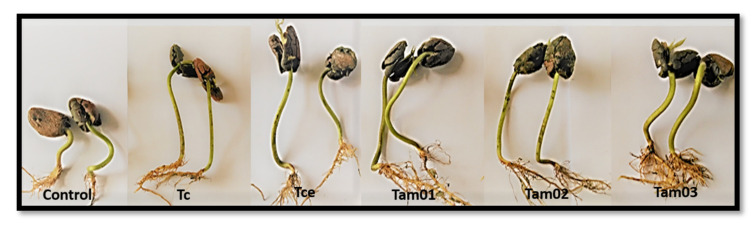
Radicle and hypocotyl lengths of cocoa seedlings resulting from seeds treated or not with different *Trichoderma* spp. isolates.

All *Trichoderma* isolates increased radicle length compared to the control (Table 1, Figure 1), ranging from 1.9 to 3.1 cm, corresponding to an increase from 158% to 258.3%, respectively. For the length of the hypocotyl, there was a significant difference except for the seeds treated with isolate Tam02, which did not differ from the control (Table 1). The increase in hypocotyl ranged from 0.9 to 1.3 cm, representing an increase from 69.2% to 100% for the Tce and Tc isolates, respectively.

The incidence of other fungi was observed only in the control treatment, with 26.5% of contaminated seeds (Table 1). They did not affect, however, the germination of seeds.

### 2.2. Trichoderma spp. in the Production of T. cacao Seedlings

A significant difference for the height and root dry mass of seedlings, at three and six months, was observed (Table 2).

**Figure 2:**
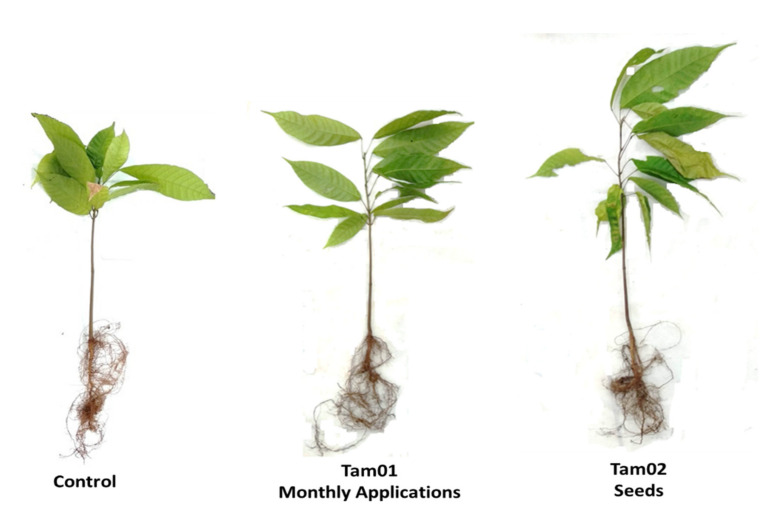
Cocoa seedlings with and without application of *Trichoderma asperellum*, six months after planting.

Two *Trichoderma* isolates increased plant height compared to the control. They were *T. asperellum* Tam01, applied monthly to the substrate after planting in the two evaluation periods, and *T. asperellum* Tam02, applied to the seeds in the evaluation at six months only (Table 2, Figure 2). The increase in height provided by Tam01 achieved 4.4 cm at three months and 5.8 cm at six months, corresponding to an increase by 17.2% and 20.4%, respectively. For Tam02 applied to seeds, the increase in height was 5.9 cm (20.7%) when compared to the control.

Tam01 also showed an increase in root dry mass of plants in the triple combination of application modes (seeds + substrate pre-planting + monthly applications in the substrate post planting) (Table 2), with an increase of 1.1 g in relation to the control, corresponding to a 64% increment.

When comparing the treatments, there was a significant difference for the interaction between (*Trichoderma* isolates × application modes) only for the plant height at six months after planting. In the mode of application via seeds, the height of seedlings treated with Tam02 was greater than those that received the application of isolates Tc and Tam03. In the monthly applications in the post-planting substrate, a difference was observed only between Tam01 and Tam02, with mean height values of 34.2 cm and 29.4 cm, respectively (Table 3).

## 3. Discussion

Biological agents applied to seeds and seedlings are important components of sustainable agriculture strategies. In this study, *Trichoderma* spp. natives of the Amazon region were used in cocoa, a naturally occurring species of this biome.

Although *Trichoderma* isolates did not increase germination parameters compared to the control, they positively affected seedlings growth. Data indicate a healthy condition of the seeds used, showing an average germination close to that of newly harvested fruits of this species, which can reach 100% [16]. This was likely due to storage lipids that act as carbon and energy reserves for embryos to germinate, favoring high germination rates [17]. On the opposite, the storage of seeds for long periods of time affects germination, as seeds do not tolerate dehydration or low temperatures [18].

Biological treatment positively influenced other variables related to cocoa seed germination, such as radicle and hypocotyl length. Data showed that the greatest increase in radicle length was 3.6-times higher than the control and twice the hypocotyl length. Indoleacetic acid (IAA) appears as a possible effector in this interaction. Root exudates are sources of the amino acid L-tryptophan, a precursor for IAA biosynthesis by the rhizosphere microbiota [19,20]. IAA regulates several stages of plant growth and development, such as cell elongation and division, tissue differentiation, and apical dominance [21]. In addition to auxins, cytokinin produced by microorganisms promote plant growth and cell division [22]. The effects observed on shoot growth may be hence related to the production active effectors translocated from the roots to the shoot and induced by auxin-producing microorganisms that thus stimulate the root system [23]. The production of auxins by *Trichoderma* spp. has already been shown [24,25].

There are several reports indicating a biostimulation effect induced by *Trichoderma* spp. Seeds of *Theobroma grandiflorum*, a relative of cocoa, treated with isolates of *T. asperellum* showed an increased length of the radicle in the seedlings but no effect on germination parameters [26]. Moreover, the incidence of fungi was observed only in untreated seeds, demonstrating the efficiency of *Trichoderma* in controlling fungi naturally occurring in cocoa seeds. Similarly, in *T. grandiflorum*, no fungi were observed in seeds treated with the same *Trichoderma* isolates herein evaluated [26].

The absence of harmful fungi in seeds is important for developing of healthy seedlings and for their survival in the field. Among seeds colonizing fungi, species of *Aspergillus* and *Penicillium* have been reported as potential producers of mycotoxins in cocoa beans [27]. Seed treatments are important to reduce or eliminate microorganisms that can harm the seeds and affect the initial development of seedlings. Additionally, since *Trichoderma* spp. includes important biocontrol agents, biological treatments appear useful to protect the young plants from the attack of pathogens already present in the soil.

Identifying the ideal conditions for seed germination is an important factor to ensure uniform production of quality seedlings and for the establishment of a standard in the field production [28]. In the nursery assay, only two *Trichoderma* isolates applied in seeds and in the post-planting substrate influenced height (Tam01 and Tam02) and root dry mass (Tam01). Differences in isolates efficiency may be related to different mechanisms of action in plant growth promotion and biological control that may vary according to environmental conditions (substrate, nutrients, other microorganisms), species, or association with the host plant [29].

Growth promotion is not a general feature for all *Trichoderma* spp., as different effects on plants have been observed. In addition, promotion variability may be related to and influenced by environmental factors [30,31]. In a collection from Colombia, 60% of *Trichoderma* strains produced IAA derivatives in vitro, but only 18% showed plant growth effects on beans [32]. In addition to this, auxins and volatile organic compounds have been described as growth-promoting inducers in fungi, including *Trichoderma* [33]. Evaluation of *Trichoderma* spp. isolates in *Jatropha curcas* and *Ricinus communis* showed negative effects for some isolates on the diameter, number of leaves, and dry mass of the aerial part and root system of plants [34]. A promotion effect on root dry mass and height were instead reported in *Gochnatia polymorpha* plants treated with *Trichoderma harzianum* [29]. Seedlings of *Handroanthus serratifolius* (yellow ipe) treated with *Trichoderma* showed increased height, stem diameter, number of leaves, and root dry mass compared to the control [35]. Increased height of plants treated with *Trichoderma* was also found in *Khaya ivorensis* and *Eucalyptus camaldulensis,* with increased number of leaves and higher shoot and root dry mass, and *Pinus radiata* [36,37,38].

The 64% increase in root dry mass observed in the treatment with *Trichoderma* Tam01 isolate and applied in a triple combination (seeds + pre-planting substrate + monthly applications in post-planting substrate) is a promising result. The plant’s investment in root growth is one of the main benefits attributed to microorganisms that produce IAA. For young plants, the growth and establishment of roots is essential, as the increase in root biomass promotes adherence to the substrate and greater absorption of water and nutrients. These effects increase nutrients accumulation and cell multiplication through the production of hormones and other metabolites, consequently increasing the chances of survival in the field [39,40,41,42,43,44,45,46].

The application of *Trichoderma* Tam01 isolate in the pre-planting substrate combined with applications in the post-planting substrate also positively influenced the development of *Euterpe oleracea* seedlings [2]. The use of *Trichoderma* in the pre-planting substrate increased the height, stem diameter, and number of leaves of *E. schomburgkii* seedlings [47]. *Trichoderma* spp. used in seeds or in combination with substrate application were also effective on seedlings of *Khaya ivorensis* [36]. In seedlings of *Parapiptadenia rigida*, the application of *Trichoderma* via seeds showed the greatest increase in plant height [48].

*Trichoderma* spp. have potential for application with several purposes, such as biological control of plant diseases, production of enzymes for industries, and other fundamental aspects, such as increasing the efficiency of nitrogen use, promoting the development of plants, as well as productivity and alleviating the impacts of salt stresses [49].

## 4. Materials and Methods

The research was registered in the National System for the Management of Genetic Heritage and Associated Traditional Knowledge (SISGEN-Brazil) under protocol A67256B. The evaluation of *Trichoderma* isolates on *T. cacao* was performed in two tests: in the laboratory, to evaluate the effect of treatment on germination and initial development of the seedlings, and in a nursery, to assess the effect on seedling development.

### 4.1. Obtaining and Mass Production of Trichoderma spp. Isolates

Five *Trichoderma* isolates were used: three belonging to the species *Trichoderma asperellum* (Tam01, Tam02, and Tam03) hailing from rhizospheric soil of forest species in reforested areas and native forests from the Urucu Base, Coari, and two species of *Trichoderma* sp. (Tc and Tce) from soils of agroforestry systems with Curauá (*Ananas erectifolius* L.B.Smith) and Cumaru (*Dipteryx odorata* (Aubl.) Willd.), from São Pedro Community region of Eixo Forte, Santarém, all from Brazilian Amazonia. In this research, we prioritized using *Trichoderma* isolates from the Amazonia, as they are adapted to the conditions of this region. All isolates are stored in the Micoteca of the Phytopathology Laboratory of the Federal University of Western Pará-Ufopa.

*Trichoderma* isolates were previously grown in Petri dishes containing potato-dextrose-agar (PDA) culture medium and mass-produced in parboiled rice for the preparation of suspensions used in germination tests and seedling production. The preparation of the suspension of each isolate occurred at the time of setting up the experiment, using distilled and sterilized water.

### 4.2. Trichoderma Effect on Germination of T. cacao Seeds

The experiment was performed at the Phytopathology Laboratory of the Ufopa. For treating T. cacao seeds, fungal suspensions were prepared with each Trichoderma isolate at a concentration of 1 × 10^8^ conidia·mL^−1^, in which the seeds were immersed for 24 h [2]. The concentration used was based on the recommendation to use *Trichoderma* in cocoa as a biocontrol agent [49]. The control treatment consisted of immersing the seeds only in distilled and sterilized water for the same time. After microbiolization, the seeds were then placed in plastic trays containing two layers of moistened filter paper and maintained at room temperature (±27 °C) during the evaluation period.

The experimental design was completely randomized (CRD), with four replications containing 50 seeds each, making 1200 seeds. We evaluated: (a) germination speed index (IVG) [50]; (b) germination, counting the number of germinated seeds, and, after three stable counts, determining the percentage of germination; (c) lengths of the radicle and hypocotyl, measuring the structures with the aid of a millimeter ruler after the germination count; and (d) incidence of fungi by counting the number of seeds with the presence of fungi under a stereoscopic microscope except for *Trichoderma* in those treatments that received the biological treatment.

### 4.3. Trichoderma spp. and Development of T. cacao Seedlings

The trial was conducted in the Forest Seedling Nursery of the Institute of Biodiversity and Forests, Ufopa, Santarém, Pará. The average annual temperature of the city of Santarém is approximately 27.2 °C, and average annual rainfall is 3109 mm [51].

*Trichoderma* isolates were tested in four application modes: (a) on seeds; (b) on the pre-planting substrate; (c) monthly in the post-planting substrate; and (d) via seeds + pre-planting substrate + monthly in post-planting substrate. Plants without *Trichoderma* application served as a control treatment. We tested different modes of application to select the most suitable method for plant growth and the greatest feasibility of execution by the farmer.

The planting of *T. cacao* seeds was conducted in polyethylene bags containing 1.5 kg of forest soil, keeping the seedlings in a nursery with 50% shading throughout the evaluation period.

For the treatments, suspensions were prepared with each of the *Trichoderma* isolates at a concentration of 1 × 10^8^ conidia·mL^−1^. The treatments that used the application of *Trichoderma* via seeds consisted of immerson in *Trichoderma* suspensions for 24 h one day before planting. Applications of *Trichoderma* in the pre-planting substrate were performed seven days before planting by first wetting the soil with 10 mL of water and then applying 10 mL of suspension of each isolate in the respective treatments. Monthly applications of *Trichoderma* isolates in the post-planting substrate began 30 days after planting, applying monthly, as in the treatment in the pre-planting substrate, until six months of seedling production. The treatments that received the combination of the three modes of application followed the same methodology adopted for each one individually.

The trial was set up in CRD, in a factorial scheme (4 × 5 + 1), with four application modes × five *Trichoderma* isolates + a control treatment, with eight replications and one plant per replication.

We evaluated monthly: (a) plant height, by measuring the base of the stem to the apex with the aid of a millimeter ruler; (b) diameter of the collar with a digital caliper; and (c) number of leaves, by counting the total number of leaves on each plant. At the end of the experiment (six months), we evaluated: (d) length of the root system, measuring the root with a millimeter ruler; (e) leaf area, using the leaf area meter AM350 (ADC Bio Scientific Ltd., Hoddesdon, UK); and (f) dry mass of the aerial part and of the root system, weighing the parts separately after drying in an oven with forced air circulation at 60 °C, for 72 h.

### 4.4. Data Analysis

Analysis of variance (ANOVA) was performed for laboratory data, and treatment means were compared by Tukey’s test (*p* ≤ 0.05). For the nursery trial, ANOVAs were performed using data obtained at three and six months, using the Dunnett test (*p* ≤ 0.05) to compare the mean of the treatments with the control and the Tukey test (*p* ≤ 0.05) to compare the means of the treatments with each other, using the statistical program Assistat^®^ version 7.7 [52].

## 5. Conclusions

Biological treatment of *Theobroma cacao* seeds had a positive effect on the development of seedling structures and fungal control. The use of *Trichoderma* spp. isolates on the seeds is the easiest and most viable way of application for the production of cocoa seedlings. This facility and the results obtained for seeds should encourage the use of *Trichoderma* by cocoa farmers in the region.

The results show a possibility of producing cocoa seedlings using a sustainable technique based on the use of biostimulants for plant production. Future studies can be conducted to verify the potential of these fungi as biostimulants of seedlings and plants in the field, with benefits for the environmental and consumers’ health.

## Figures and Tables

**Table 1:** Germination and germination speed index (SG) and radicle and hypocotyl lengths of *Theobroma cacao* seeds treated or not with different *Trichoderma* isolates. Incidence of fungi represent the % of seed contaminated with other fungi.

Treatment	Germination (%)		SG		Radicle (cm)		Hypocotyl (cm)		Incidence of Fungi (%)
Control	97.0	ab	58.3	a	1.2	c	1.3	c	26.5
*Trichoderma* sp. Tc	98.0	a	61.7	a	4.3	a	2.6	a	0.0
*Trichoderma* sp. Tce	97.5	b	61.7	a	4.2	a	2.2	ab	0.0
*T. asperellum* Tam01	96.5	ab	61.9	a	4.1	a	2.3	ab	0.0
*T. asperellum* Tam02	98.0	a	54.4	a	3.2	b	1.6	bc	0.0
*T. asperellum* Tam03	95.0	b	52.6	a	3.1	b	2.4	a	0.0
CV(%)	1.3		7.7		9.8		15.8		

CV, coefficient of variation; means followed by the same letters in the columns do not differ from each other by the Tukey test (*p* ≤ 0.05).

**Table 2:** Height (cm), diameter of the collar (mm), number of leaves per plant, dry mass (DM) of aerial shoot and root system (g), root length (cm), and leaf area (cm^2^) of *Theobroma cacao* seedlings submitted to different application modes and *Trichoderma* spp. isolates.

Treatments	Height		Collar Diameter		Number of Leaves		Aerial DM	Root DM	Root Length	Leaf Area
	3rd month	6th month	3rd month	6th month	3rd month	6th month				
Tc applied to seeds	26.0 ^ns^	27.9 ^ns^	5.4 ^ns^	6.2 ^ns^	7.7 ^ns^	9.3 ^ns^	2.8 ^ns^	1.9 ^ns^	49.1 ^ns^	613.1 ^ns^
Tc applied to pre-planting substrate	24.9 ^ns^	29.6 ^ns^	5.2 ^ns^	6.4 ^ns^	7.5 ^ns^	10.6 ^ns^	3.3 ^ns^	2.0 ^ns^	54.4 ^ns^	437.7 ^ns^
Tc applied monthly to post-planting substrate	25.7 ^ns^	30.3 ^ns^	5.7 ^ns^	6.5 ^ns^	7.8 ^ns^	12.7 ^ns^	4.1 ^ns^	2.2 ^ns^	47.0 ^ns^	613.1 ^ns^
Tc seeds + substrate + monthly	28.2 ^ns^	32.3^ns^	5.5 ^ns^	6.3 ^ns^	8.8 ^ns^	11.2 ^ns^	3.3 ^ns^	2.1 ^ns^	54.9 ^ns^	448.7 ^ns^
Tce applied to seeds	27.0 ^ns^	30.4 ^ns^	5.2 ^ns^	6.4 ^ns^	7.2 ^ns^	10.3 ^ns^	3.6 ^ns^	2.3 ^ns^	51.1 ^ns^	551.8 ^ns^
Tce applied to pre-planting substrate	26.5 ^ns^	31.2 ^ns^	5.2 ^ns^	6.2 ^ns^	8.0 ^ns^	10.2 ^ns^	3.0 ^ns^	1.8 ^ns^	51.7 ^ns^	539.1 ^ns^
Tce applied monthly to post-planting substrate	24.5 ^ns^	30.6 ^ns^	5.0 ^ns^	6.2 ^ns^	8.8 ^ns^	11.1 ^ns^	3.4 ^ns^	2.0 ^ns^	54.9 ^ns^	438.6 ^ns^
Tce seeds + substrate + monthly	26.0 ^ns^	31.1 ^ns^	5.4 ^ns^	6.3 ^ns^	9.0 ^ns^	10.5 ^ns^	3.6 ^ns^	2.3 ^ns^	52.8 ^ns^	413.6 ^ns^
Tam01 applied to seeds	26.9 ^ns^	32.3 ^ns^	5.4 ^ns^	6.7 ^ns^	8.5 ^ns^	11.0 ^ns^	3.6 ^ns^	1.9 ^ns^	52.3 ^ns^	514.1 ^ns^
Tam01 applied to pre-planting substrate	26.8 ^ns^	29.8 ^ns^	5.1 ^ns^	6.0 ^ns^	8.0 ^ns^	10.6 ^ns^	2.8 ^ns^	1.9 ^ns^	53.4 ^ns^	380.6 ^ns^
Tam01 applied monthly to post-planting substrate	30.0 *	34.2 *	5.0 ^ns^	6.5 ^ns^	8.7 ^ns^	11.8 ^ns^	3.8 ^ns^	1.8 ^ns^	50.0 ^ns^	585.5 ^ns^
Tam01 seeds + substrate + monthly	27.6 ^ns^	31.5 ^ns^	5.8 ^ns^	6.6 ^ns^	9.5 ^ns^	11.1 ^ns^	3.4 ^ns^	2.8 *	55.2 ^ns^	559.2 ^ns^
Tam02 applied to seeds	27.9 ^ns^	34.3 *	5.2 ^ns^	6.3 ^ns^	8.8 ^ns^	11.2 ^ns^	3.4 ^ns^	1.7 ^ns^	59.6 ^ns^	529.9 ^ns^
Tam02 applied to pre-planting substrate	25.7 ^ns^	31.4 ^ns^	5.2 ^ns^	6.2 ^ns^	8.6 ^ns^	11.5 ^ns^	3.9 ^ns^	2.1 ^ns^	61.9 ^ns^	491.4 ^ns^
Tam02 applied monthly to post-planting substrate	27.3 ^ns^	29.4 ^ns^	5.1 ^ns^	6.1 ^ns^	7.8 ^ns^	9.2 ^ns^	2.9 ^ns^	1.6 ^ns^	53.1 ^ns^	442.2 ^ns^
Tam02 seeds + substrate + monthly	28.8 ^ns^	32.4 ^ns^	5.5 ^ns^	6.5 ^ns^	8.8 ^ns^	9.6 ^ns^	3.5 ^ns^	2.1 ^ns^	56.1 ^ns^	464.0 ^ns^
Tam03 applied to seeds	25.8 ^ns^	28.3 ^ns^	5.3 ^ns^	6.2 ^ns^	7.8 ^ns^	9.6 ^ns^	3.2 ^ns^	2.0 ^ns^	43.6 ^ns^	494.4 ^ns^
Tam03 applied to pre-planting substrate	26.5 ^ns^	30.2 ^ns^	5.3 ^ns^	6.3 ^ns^	7.2 ^ns^	10.3 ^ns^	2.8 ^ns^	1.5 ^ns^	53.2 ^ns^	425.4 ^ns^
Tam03 applied monthly to post-planting substrate	27.0 ^ns^	29.9 ^ns^	5.2 ^ns^	6.0 ^ns^	7.7 ^ns^	10.7 ^ns^	3.3 ^ns^	1.7 ^ns^	51.1 ^ns^	422.8 ^ns^
Tam03 seeds + substrate + monthly	26.8 ^ns^	31.1 ^ns^	5.5 ^ns^	6.4 ^ns^	7.8 ^ns^	9.2 ^ns^	3.1 ^ns^	1.9 ^ns^	56.4 ^ns^	385.1 ^ns^
Control	25.6	28.4	5.1	6.0	7.6	10.7	3.4	1.7	51.5	484.1
Coefficient of Variation (%)	9.9	10.5	10.4	11.4	18.6	22.1	28.0	31.9	23.2	20.7

* Significant; ns, not significant; by Dunnett test (*p* < 0.05). Tc, *Trichoderma* sp.; Tce, *Trichoderma* sp.; Tam01, *T. asperellum;* Tam02, *T. asperellum;* Tam03, *T. asperellum.*

**Table 3:** Height of *Theobroma cacao* seedlings in relation to different modes of application modes of *Trichoderma* spp., six months after planting.

Modes of Application	*Trichoderma* Isolates									
	Tc		Tce		Tam01		Tam02		Tam03	
Seeds ^1^	27.9	bB	30.3	aAB	32.3	abAB	34.3	aA	28.2	aB
Substrate ^2^	29.5	abA	31.2	aA	29.7	bA	31.3	abA	30.2	aA
Monthly Aplication ^3^	30.3	abAB	30.6	aAB	34.2	aA	29.4	bB	29.9	aAB
Seed + Subs. + Mon.Ap. ^4^	32.3	aA	31.0	aA	31.5	abA	32.4	abA	31.1	aA
CV (%) 10.5										

Means followed by the same lowercase letters in the columns and the same uppercase letters in the rows do not differ by Tukey’s test (*p* ≤ 0.05). ^1^ = *Trichoderma* application to seeds; ^2^ = application of *Trichoderma* in the substrate at pre-planting; ^3^ = monthly application of *Trichoderma* in the pos-planting substrate; ^4^ = *Trichoderma* applied seeds + application in the substrate at pre-planting + monthly application in the substrate post planting.

## Data Availability

The data presented in this study are available in the text, figures, and tables.
